# Subjective Impairments in Olfaction and Cognition Predict Dissociated Behavioral Outcomes

**DOI:** 10.1093/geronb/gbac124

**Published:** 2022-08-24

**Authors:** Nira Cedres, Andrea Aejmelaeus-Lindström, Ingrid Ekström, Steven Nordin, Xin Li, Jonas Persson, Jonas K Olofsson

**Affiliations:** Department of Psychology, Sensory Cognitive Interaction Laboratory (SCI-Lab), Stockholm University, Stockholm, Sweden; Division of Clinical Geriatrics, Centre for Alzheimer Research, Department of Neurobiology, Care Sciences, and Society, Karolinska Institutet, Stockholm, Sweden; Department of Psychology, Faculty of Health Sciences, University Fernando Pessoa Canarias, Las Palmas de Gran Canaria, Spain; Department of Psychology, Sensory Cognitive Interaction Laboratory (SCI-Lab), Stockholm University, Stockholm, Sweden; Aging Research Center, Department of Neurobiology, Care Sciences and Society, Karolinska Institutet, Stockholm, Sweden; Department of Psychology, Umeå University, Umeå, Sweden; Aging Research Center, Department of Neurobiology, Care Sciences and Society, Karolinska Institutet, Stockholm, Sweden; Aging Research Center, Department of Neurobiology, Care Sciences and Society, Karolinska Institutet, Stockholm, Sweden; Department of Psychology, Sensory Cognitive Interaction Laboratory (SCI-Lab), Stockholm University, Stockholm, Sweden

**Keywords:** Anosmia, Cognition, Subjective cognitive decline, Subjective olfactory impairment, Olfaction

## Abstract

**Background:**

Self-rated subjective cognitive decline (SCD) and subjective olfactory impairment (SOI) are associated with objective cognitive decline and dementia. However, their relationship and co-occurrence is unknown. We aimed to (a) describe the occurrence of SOI, SCD and their overlap in the general population; (b) compare SOI and SCD in terms of longitudinal associations with corresponding objective olfactory and cognitive measures; and (c) describe how SOI and SCD may lead to distinct sensory and cognitive outcomes.

**Methods:**

Cognitively unimpaired individuals from the third wave of the Swedish population-based Betula study (*n* = 784, aged 35–90 years; 51% females) were split into self-rated SOI, SCD, overlapping SCD + SOI, and controls. Between-subject and within-subject repeated-measures MANCOVA were used to compare the groups regarding odor identification, cognition, age, sex, and education. Spearman correlation was used to assess the different patterns of association between olfaction and cognition across groups.

**Results:**

SOI was present in 21.1%, whereas SCD was present in 9.9% of participants. According to a chi-square analysis, the SCD + SOI overlap (2.7%) is on a level that could be expected if the phenomena were independent. Odor identification in SOI showed decline at the 10-year follow-up (*n* = 284) and was positively associated with cognition. The SOI and SCD groups showed distinct cognitive-olfactory profiles at follow-up.

**Conclusions:**

SOI occur independently of SCD in the population, and these risk factors are associated with different cognitive and olfactory outcomes. The biological causes underlying SOI and SCD, as well as the risk for future cognitive impairment, need further investigation.

Age-related disease and comorbidities results in poor quality of life and increasing economic costs for society (World Health Organization [WHO)., 2020]. As the aging population is increasing globally, development of cost–effective tools to identify individuals at risk, and reducing the associated social and economic impact has become a prioritized goal (Golubnitschaja et al., 2014). In particular, self-rated reports about health status are useful in predicting future disease outcomes and mortality (DeSalvo et al., 2006; Jylhä et al., 2006). Notably, self-rated health reports often outperform objective assessments in this regard and may provide flexible, low-cost screening tools (Slot, Sikkes, Berkhof, Brodaty, van der Flier, et al., 2019). For this reason, self-rated health is widely used in a variety of settings (Aichele et al., 2016; Castellon et al., 2004; Jylhä et al., 2006; Österberg et al., 2014; Suhr, 2003; Zamarian et al., 2015).

The present study focused on comparing and contrasting self-reported impairments in cognition and the sense of smell, olfaction. Cognitive decline is the primary preclinical expression of dementia, which is associated with a global annual cost of 948 U.S. billion dollars (Xu et al., 2017). Subjective cognitive decline (SCD) is often reported even though a formal neuropsychological evaluation shows no cognitive impairment (Jessen, Amariglio, van Boxtel, Breteler, Heun, et al., 2014), and this have proven to be specially relevant in individuals actively seeking for help (Jessen et al., 2020). On the other hand, the presence of SCD has been associated with subclinical changes in functionality in community-based samples with low frequency of help-seeking behaviors (Cedres et al., 2019). Yet, SCD individuals have shown an increased risk for *future* cognitive impairment and dementia compared to non-SCD, independent of the origin of the sample (i.e., memory clinic or general population; Slot, Sikkes, Berkhof, Brodaty, van der Flier, et al., 2019). Furthermore, SCD has been associated with multiple underlying brain pathological markers, such as increased amyloidosis and tauopathies (Buckley et al., 2017), gray and white matter neurodegeneration (Cedres et al., 2021), and small vessel disease (Diaz-Galvan, Cedres, et al., 2021). In fact, it has been shown that amnestic SCD (i.e., cognitive complaints referring to the memory domain) presents a brain atrophy pattern similar to that typically presented in Alzheimer’s disease (AD; Diaz-Galvan, Ferreira, et al., 2021). On the other hand, the same study showed that nonamnestic SCD seems to be associated with increased underlying small vessel disease in the general population (Diaz-Galvan, Ferreira, et al., 2021).

Subjective olfactory impairment (SOI) may provide a complementary assessment of brain health in older adults, as it concerns the self-reported inability to perceive smells. SOI is associated with an increased risk of future dementia and mortality—even after controlling for both cognitive and objective olfactory performance levels (Ekström et al., 2017). Despite these results, SOI has been questioned since it is not strongly correlated with objective performance levels in olfactory tests (Wehling et al., 2011). However, neither self-reported cognitive measures are highly correlated with objective performance levels (Cedres et al., 2019), yet, these subjective reports have a strong predictive utility, arguably because they accurately capture perceived intra-individual declines (DeSalvo et al., 2006; Jonker et al., 2000; Slot, Sikkes, Berkhof, Brodaty, van der Flier, et al., 2019). Since the start of the COVID-19 pandemic, self-rated olfaction has become a widely adopted assessment tool (Pallanti, 2020), and it was proposed as the first step in the current screening guidelines for olfactory dysfunction (Boesveldt et al., 2017). Olfactory impairment in older adults is associated with cognitive decline and atrophy in the medial temporal lobe (Dintica et al., 2019; Olofsson et al., 2016; Schubert et al., 2008), but the association between SOI and SCD is not yet understood (Jobin, Zahal, et al., 2021).

SOI may provide a useful and cost-efficient tool to understand and predict trajectories in cognitive brain aging, but as noted above, its role is controversial. Although both SCD and SOI are frequently reported in the population, it is unclear as to what extent they co-occur and whether individuals reporting both symptoms are at an especially increased risk for future cognitive decline and/or olfactory dysfunction. If the overlap is high, SOI might be just a reflection of general self-awareness (i.e., people who report cognitive decline may report other types of decline as well, without providing an accurate olfactory self-report), and SOI could then be disregarded. If, on the other hand, SOI affects different individuals than SCD, and is associated with distinct and meaningful outcomes, that would strengthen the role of SOI as a useful assessment of brain health during aging. Our aim was thus to describe the occurrence of SOI, SCD, and their overlap in the general population. Furthermore, we aimed to determine how SOI and SCD are associated with objectively assessed olfaction and cognition at baseline and at a 10-year follow-up. Finally, we describe the differential patterns of association between objectively assessed olfaction and cognitive performance (global cognition, memory, verbal fluency, and visuospatial abilities) across SOI, SCD, and healthy controls.

## Method

### Participants

The study sample belongs to the Betula project (Nilsson et al., 1997; Rönnlund et al., 2011), a large-scale population-based longitudinal cohort from Umeå, Sweden available on request. Betula project data collection has been completed at six occasions (1988–1990, 1993–1995, 1998–2000, 2003–2005, 2008–2010, and 2013–2014). The project includes data on participants’ sociodemographic features and several cognitive, psychological, and physical health variables. Every person over 30 years whose age ended in 0 or 5 (i.e., 30, 35, 40, 45, etc.) entered in the pool for random selection (*n* = 100 at each age group) based on the Umeå population registry (85% initial response rate; Nilsson et al., 1997). The general examinations and other procedures have been described in detail previously (Nilsson et al., 1997; Rönnlund et al., 2011). From the second data collection onwards, additional samples were collected following the same recruitment procedure to compensate for learning effects and incident death in the original sample. Olfactory assessment was included from the third data collection onwards. Thus, the third time point (i.e., T3, 1998–2000), including samples S1 and S2, was used as baseline assessment for the present study, and the fifth time point (i.e., T5, 2008–2010), including solely S1 participants, as 10-year follow-up. S2 data collection was discontinued, and these participants thus provided no follow-up data. Further details on Betula study design have been explained elsewhere (Nilsson et al., 1997; Rönnlund et al., 2011). The fourth data collection (i.e., T4, 2003–2005) was not considered for the longitudinal analysis in the current study, since little change is expected after 5 years in cognitively healthy individuals from the general population. To further support this decision, the presence of statistically significant differences was tested between baseline (i.e., T3 time point) and T4 time point and presented in the Results section. The use of a 10-year follow-up was expected to increase our statistical power.

For the current study, inclusion criteria were as follows: (a) preserved global cognition and functionality, operationalized as a Mini-Mental State Examination (MMSE; Folstein et al., 1975) score ≥24, and a Betula Activities of Daily Living (ADL) Index score = 1 (i.e., independent; Nilsson et al., 1997); (b) no medical history of neurological and psychiatric disorders (including a diagnosis of major depression), systemic diseases or head trauma; (c) no history of substance abuse; and (d) the absence of missing values in the study variables of interest, that is, self-rated sense of smell (*n* = 17), self-rated cognitive performance (*n* = 6), objective olfactory performance (*n* = 19), yielding a final baseline sample of 784 individuals (51% females) selected from all the 2466 available participants.

Participants’ written consent was obtained in accordance with the Declaration of Helsinki (BMJ 1991; 302: 1194) and the study was approved by the Regional Ethical Vetting Board at Umeå University and the Swedish Ethical Review Authority (approval numbers 870303, 97-173, 221/97, 97-173, 03-484, 01-008, 169/02, 02-164, 05-082M, and 08-132M).

### SCD and SOI Assessment

SCD was operationalized accordingly with the SCD Initiative (SCD-I) guidelines (Jessen, Amariglio, van Boxtel, Breteler, Ceccaldi, et al., 2014). Participants were asked a single Likert scale question referring to each individual’s self-rated episodic memory performance (Table 1). Participants reporting memory as “much worse” or “somewhat worse” were categorized as SCD. Likewise, SOI was operationalized based on a single question referring to each individual self-rated olfactory performance (Table 1). Participants reporting olfaction as “no ability” or “worse than normal” were categorized as SOI, whereas “normal” or “better than normal” was categorized as no SOI (similar to Ekström et al., 2017; Stanciu et al., 2014). SOI was assessed prior to the objective olfactory assessment for all participants, in order to not confound the subjective reports.

**Table 1.** Subjective Complaints Questions for SOI and SCD Group Operationalization

SCD: How do you think your memory is today compared to 5 years ago?	
SCD	1: Much worse
	2: Somewhat worse
Non-SCD	3: Same
	4: Somewhat better
	5: Much better
SOI: How is your ability to smell odors?	
SOI	0: No ability
	1: Worse than normal
Non-SOI	2: Normal
	3: Better than normal

*Notes:* SCD = subjective cognitive decline; SOI = subjective olfactory impairment.

Participants categorized as both SCD and SOI were identified in the overlapping SCD + SOI group. Finally, those categorized as neither SCD or SOI were identified as healthy controls. Note that the latter group was not screened for other health impairments, so they are healthy controls only in the sense of having neither SCD or SOI.

### Objective Olfaction and Cognition Assessment

To obtain approximately normal distributions, a revised version of the original, clinical version of the Scandinavian Odor Identification Test (SOIT; Bende & Nordin, 1997) was used to assess objective olfaction performance (Larsson et al., 2004). The SOIT is part of the Betula testing battery and includes 13 household odors to properly identify among four different alternatives. The sum score of correct answers (i.e., scores 0–13) was used to assess olfactory ability.

Global cognition was assessed based on MMSE (Folstein et al., 1975) ranging from 24 to 30 given the sample inclusion criteria. Furthermore, the association between cognition and olfaction was evaluated using bivariate Pearson correlation based on a larger selection of neuropsychological tests from the Betula battery protocol including verbal memory (i.e., delayed recall of previously presented verbs and substantives), verbal fluency (i.e., number of words recalled after a key starting-letter is presented), and visuospatial abilities (i.e., Weschler Adult Intelligence Scale-III [WAIS-III] block-design subtest; Nilsson et al., 1997).

### Statistical Analysis

Statistical analyses were conducted using the R statistical software (http://www-R-project.org). A *p*-value < .05 (two-tailed) was our criterion for significance in all analyses.

To report the distribution of SCD and SOI in the sample, frequencies and descriptive analyses were performed. An ANCOVA including sex, allergies, age, and education as covariates and chi-square analyses were used to compare the study groups baseline characteristics. Within-subject MANCOVA including sex, allergies, age, and education as covariates was applied in order to compare baseline-to-follow-up performances of the longitudinal sample between T3 and T4 time points and T3 and T5 time points. All of the MANCOVA models were run as backward stepwise analyses for covariate inclusion. Bonferroni correction was use to correct for multiple comparisons. Lastly, bivariate Spearman correlation, controlling for the effect of age and years of education, was applied to assess the different patterns of association between objective olfaction and cognition across groups, both at baseline and follow-up.

## Results

### Baseline Characteristics

**Figure 1.**
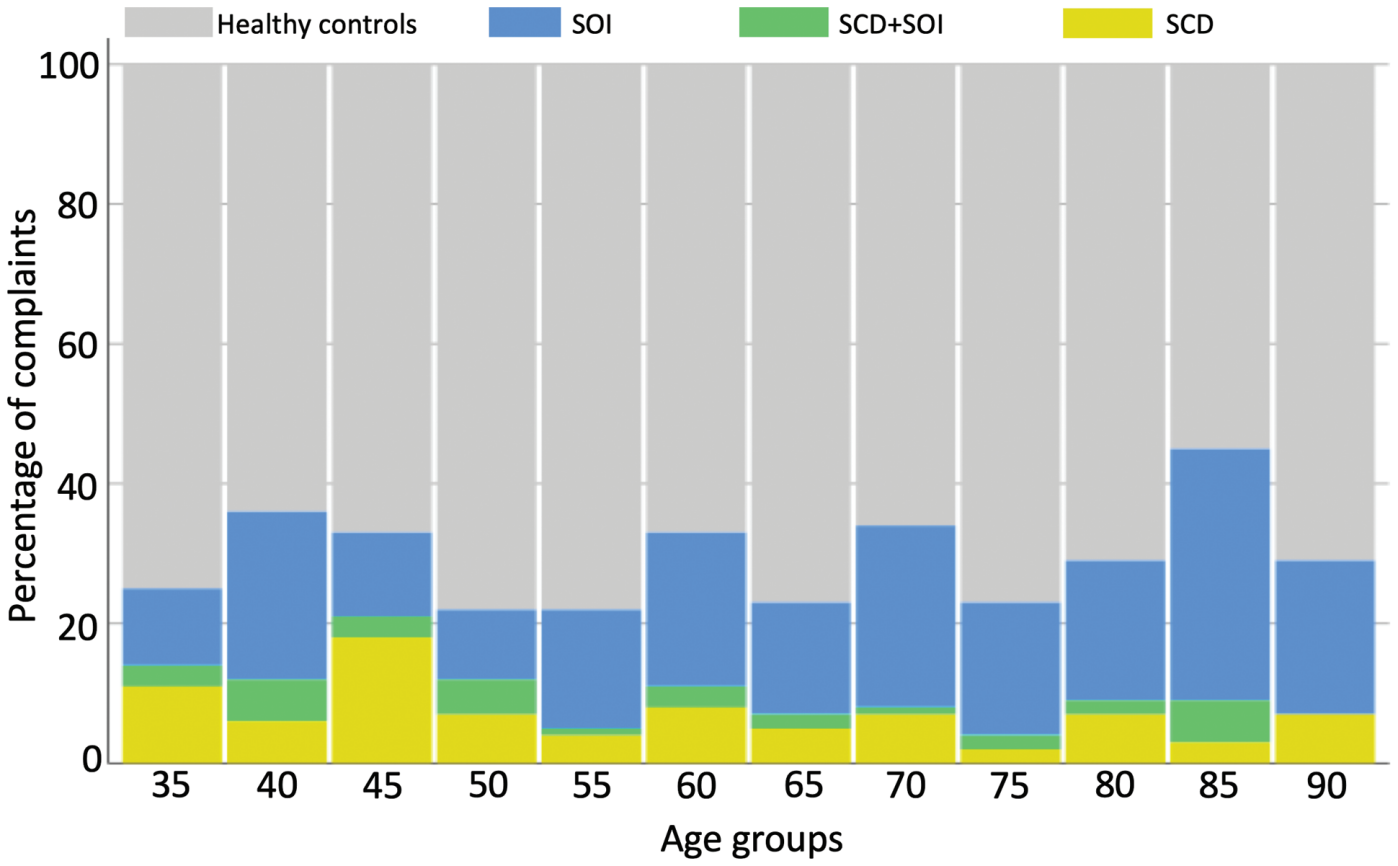
Distribution of reports of subjective cognitive decline and subjective olfactory impairment across age groups in a general sample. SOI = subjective olfactory decline; SCD = subjective cognitive decline; SCD + SOI: both SOI and SCD. X-axis represents study sample age groups from 35 to 90 years old. Y-axis represents the study groups percentages at each age.

Demographic and clinical data are given in Table 2A. From the 784 participants at baseline (i.e., T3 time point), 480 were collected from S1 and 304 were collected from S2. First, we assessed frequency and overlap of SOI and SCD in our sample; SOI was present in 21.3% (*n* = 167), whereas SCD was present in 10% (*n* = 79) of participants in the whole sample. Only 2.8% (*n* = 22) of participants reported both SOI and SCD (i.e., SCD + SOI). Relative to each group, 27.8% of the SCD individuals also reported SOI, whereas only 13% of SOI also reported SCD. The two complaints had a largely different age distribution. Visual inspection revealed that SOI was present in all age groups, yet with an increased frequency in older individuals, whereas SCD was reported mostly during middle age (Figure 1, Table 2A). Results from a chi-square analysis shows that SOI and SCD do not show increased probability of overlapping compared with what would be expected by chance (χ ^2^_1,784_ = 2.32; *p* = .146).

**Table 2.** Characteristics of the Baseline and Longitudinal Samples

		(A) Baseline sample					(B) Longitudinal sample		
	Overall	Controls	SOI	SCD	SCD + SOI	Overall	Controls	SOI	SCD
*n*	784	560	145	57	22	283	210	47	26
Age	60.84 (13.9)	60.5 (13.6)	64 (14.2)	57.3 (14)	57.3 (14.6)	68.1 (9.6)	68.9 (10.26)	70.1 (9.6)	66.9 (9.3)
Age range	35–90	35–90	35–90	35–80	35–80	55–100	55–100	55–90	55–85
Education	11.1 (4.3)	11 (4.3)	11.2 (4.5)	11.9 (3.7)	11.5 (4.7)	12.35 (4.3)	11.82 (4.4)	12.86 (4.6)	12.61 (3.6)
Sex (%female)	50.8	55.2	38.6	50.9	18.2	51.6	56.8	36.2	48.1
% Allergies	15.1	11.6	25.5	12.5	40.9	14.1	12.0	25.5	12.0
MMSE	27.95 (1.5)	27.97 (1.5)	27.91 (1.6)	28.07 (1.7)	27.45 (1.2)	28.28 (1.4)	27.64 (1.9)	27.38 (3.8)	27.59 (1.4)
SOIT	7.44 (2.2)	7.55 (2.1)	6.89 (2.4)	8.26 (2.1)	6.18 (2.5)	7.97 (1.8)	6.59 (2.4)	5.68 (2.4)	6.31 (1.8)

*Notes:* MMSE = Mini-Mental State Examination; SCD = subjective cognitive decline; SCD + SOI = both reporting SOI and SCD; SOIT = Scandinavian Odor Identification Test; SOL = subjective olfactory impairment. Values represent each variable mean and *SD*. Age and education are operationalized in years.

**Figure 2.**
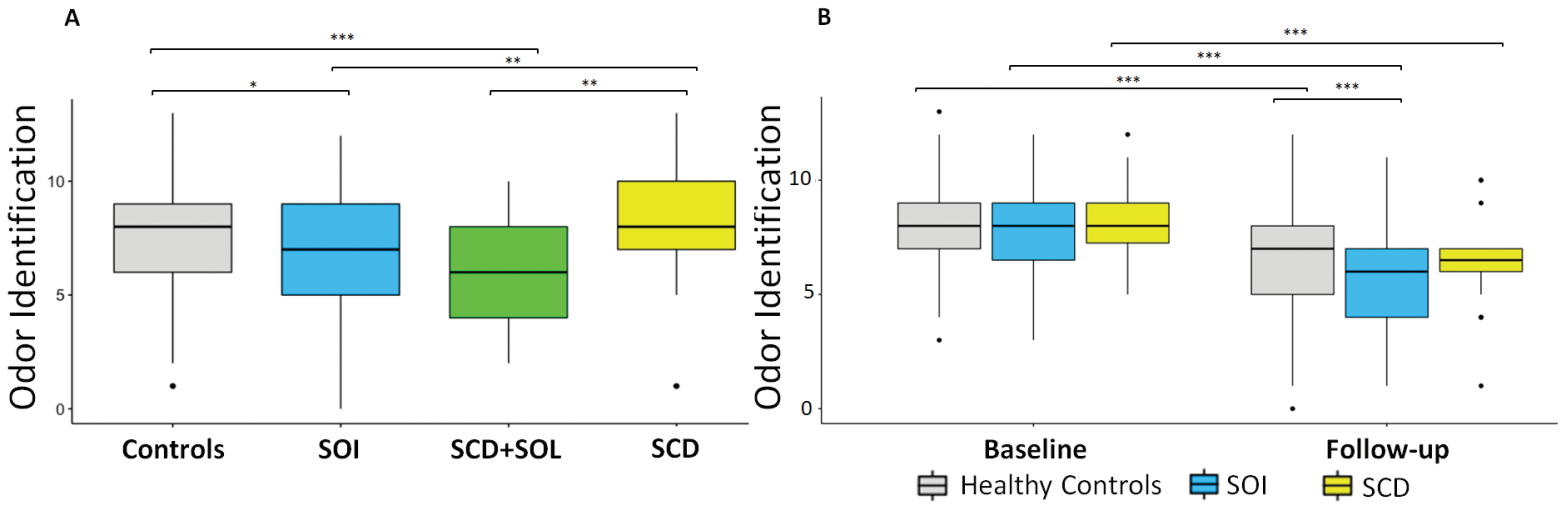
Longitudinal trajectories in odor identification and cognitive performance across and within groups. (A) Olfactory performance comparisons. (B) Cognitive performance comparisons. SCD = subjective cognitive decline ; SOI = subjective olfactory impairment. *p < .05; **p < .01; ***p < .001.

Second, we assessed baseline differences in SOIT and MMSE for the SOI, SCD, SCD + SOI, and healthy controls groups, using ANCOVA run as backwards stepwise analysis for covariates inclusion. The group comparison of SOIT scores resulted in a significant effect (*F*_3,775_ = 5.988; *p* < .001; η ^2^ = 0.023) showing that the combined SCD + SOI group had significantly lower SOIT scores compared with control (*p* = .005) and SCD groups (*p* < .001; Figure 2A). The SOI group showed significantly lower SOIT scores compared to SCD (*p* = .003) and controls (*p* = .033). Although females performed significantly better in SOIT compared with males (*F*_1,775_ = 2.277; *p* < .001; η ^2^ = 0.037), there was no significant interaction between sex and the study groups (*F*_3,775_ = 8.859; *p* = .462; η ^2^ = 0.003). Only age and level of education remained as significant covariates. Regarding global cognition measured by MMSE, the groups were not significantly different (*F*_3,775_ = 1.139; *p* = .333; η ^2^ = 0.004).

### Follow-up Characteristics

Third, we investigated future outcomes following a 5-year (*n* = 397) and a 10-year delay. After baseline data collection (T3 time point), data from S2 participants were not collected in the Betula study. Thus, only data from S1 were available for longitudinal samples. Within-subject MANCOVA between T3 and T4 time points demonstrated that none of the groups showed statistically significant differences in MMSE (*F*_2,390_ = 0.006; *p* = .999; η ^2^ < 0.001) or SOIT (*F*_2,362_ = 2.03; *p* = .109; η ^2^ = 0.017) at 5-year follow-up, supporting the usage of a longer follow-up period (i.e., 10-year follow-up, T5) when testing for longitudinal changes.

A subset of 284 individuals completed the follow-up assessment at the T5 time point, 10 years after baseline (Table 2B). As was expected, individuals in the longitudinal subset were younger (*F*_1,776.29_ = 21.18; *p* < .001) and had higher education level (*F*_1,774_ = 39.83; *p* < .001) compared with those without follow-up assessment. This indicates that older and less educated individuals (older age and lower education level are correlated in this population) are more likely to not be available for follow-up testing. As only eight participants from the combined SCD + SOI group underwent follow-up assessment, the SCD + SOI group was excluded from the longitudinal analyses due the small sample size.

As expected, within-subject MANCOVA showed a decline in SOIT scores (*F*_1,277_ = 54; *p* < .001; η ^2^ = 0.163) that was dependent on the study group (*F*_2,277_ = 54; *p* = .004; η ^2^ = 0.039; Figure 2B). Overall, participants significantly declined in MMSE scores (*F*_1,278_ = 21.3; *p* < .001; η ^2^ = 0.043), independently of the study group (*F*_2,278_ = 1.287; *p* = .278; η ^2^ = 0.009). Only level of education remained as significant covariate in both models. Interestingly, when comparing study groups at follow-up, the SOI group had significantly lower SOIT scores compared with controls (*F*_2,277_ = 5.2; *p* < .001; η ^2^ = 0.047; Figure 2B), whereas regarding cognition, none of the groups showed statistically significant differences in MMSE at follow-up (*F*_2,278_ = 0.61; *p* = .266; η ^2^ = 0.004).

### Group-Specific Associations Between Objective Olfaction and Cognition at Follow-up

**Figure 3.**
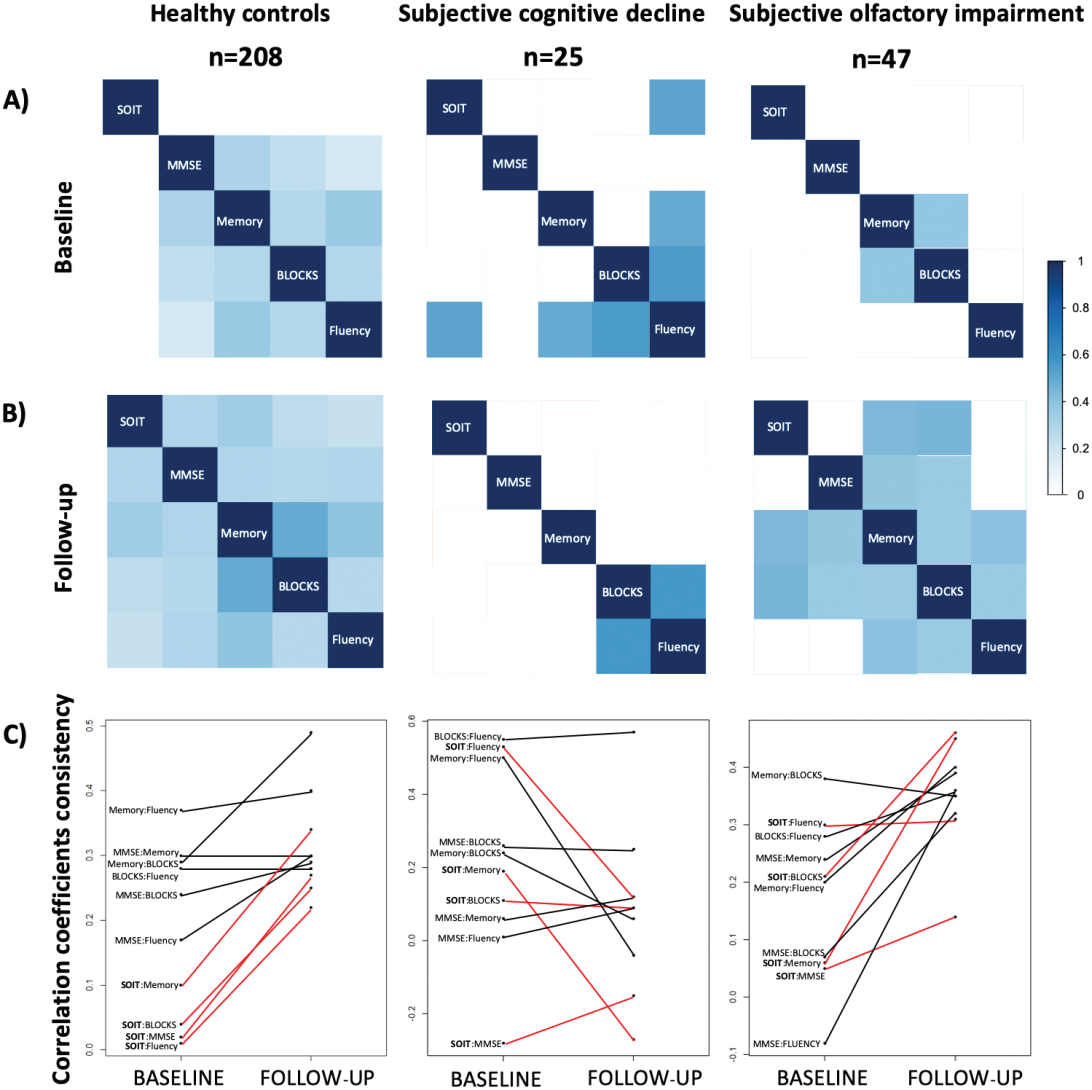
Associations between objective olfaction and cognitive measures across groups at baseline and 10-year follow-up. (A) Spearman correlation between olfaction and cognitive outcomes at baseline; (B) Spearman correlation between olfaction and cognitive outcomes at follow-up; (C) Consistency of correlation coefficients between baseline and follow-up. Red lines represent the associations of SOIT with cognitive outcomes and black lines represent the associations between cognitive outcomes; SOIT = Scandinavian Odor Identification Test; WAIS-III = Weschler Adult Intelligence Scale-III; MMSE = Mini-mental state examination; Memory = free recall of verbs and nouns; Blocks = WAIS-III Block-Design; Fluency = verbal fluency-letter A. Only significant correlation coefficients at *p* < .05 are represented. Correlations are represented in a scale from white-to-dark blue (i.e., no correlation/white; 1, positive correlation/dark blue).

We asked whether the SOI, SCD, and controls had different patterns of correlations among behavioral tests at baseline and follow-up assessments. Of particular interest was whether SOI resulted in a different emerging pattern of correlations, compared with SCD; this would strengthen the case for SOI as a predictor of cognitive brain aging. Indeed, the thre groups showed different emerging association patterns among olfaction and cognitive variables. At baseline, the healthy control group showed significant positive associations among all cognitive outcomes, but no significant associations between olfaction (SOIT) and cognition (Figure 3A). At follow-up, the control group showed significant associations between every cognitive outcome and olfaction (Figure 3B). All of the correlation coefficients increased or remained stable at follow-up for controls (Figure 3C). Of particular interest, SCD and SOI groups displayed correlation matrices that were different from that of healthy controls, and from each other, both at baseline and follow-up. The correlation coefficients in the SCD group decreased or remained relatively stable from baseline-to-follow-up (Figure 3C). Specifically, the SCD group showed a significant association between verbal fluency and objective olfaction at baseline (Figure 3A), that decreased and was no longer significant at follow-up (Figure 3B and Figure 3C). Regarding the cognitive associations in SCD, verbal fluency was also strongly positively correlated with memory and block-design at baseline (Figure 3A), but only the association between verbal fluency and block-design remained significant at follow-up (Figure 3B). The SOI group showed an opposite pattern; only memory and block-design were correlated at baseline (Figure 3A). However, at follow-up, objective olfaction (SOIT) showed significant, strong positive correlations with memory and block-design in SOI (Figure 3B). Among cognitive assessments, significant, positive correlations emerged for memory with global cognition, block-design and verbal fluency, and for block-design with global cognition and verbal fluency (Figure 3B). Overall, the correlation coefficients in the SOI group increased (Figure 3C). This indicates that SOI at baseline is not only associated with poor olfactory performance at follow-up, but also that these olfactory scores are associated with several non-olfactory cognitive scores; an indication of an accelerated loss of both olfaction and cognition in some individuals who reported SOI at baseline.

## Discussion

In this study, we assessed the relevance of SOI as a predictor of age-related impairments in olfaction and cognition, contrasting SOI from SCD. Using a large population-based sample with a 10-year follow-up, we investigated the occurrence of SOI, SCD, and their overlap in the general population. We found that SOI and SCD were rarely co-occurring. SOI was more frequently reported than SCD, especially in older adults. These findings point to SOI being a potentially useful self-report measure to predict future cognitive performance and olfactory impairment. Furthermore, we found different trajectories in SCD and SOI individuals in terms of cognition and odor identification over a 10-year follow-up. We found that SOI individuals had a steeper decline in odor identification until the follow-up, such that they were significantly worse compared with healthy controls. Thus, we propose that SOI may indicate subtle olfactory deficits that might only later become observable in objective tests.

SCD has been linked to slightly impaired odor identification abilities, according to a recent meta-analysis (Jobin, Zahal, et al., 2021). Although the participants who reported exclusively SCD did not show olfactory decline in our sample, the small group of individuals that reported SCD + SOI showed worse objective olfactory performance compared to both control and SCD groups. These results thus point to individuals with combined SCD + SOI as a specific group with increased risk for concurrent olfactory dysfunction. The lack of association between SCD and objective olfaction, while present in the SCD + SOI group, might point to different underlying pathologies for each subgroup. In this population-based cohort, we hypothesized that the exclusive SCD presentation might represent an underlying subclinical pathology, presumably from a vascular origin (Cedres et al., 2019; Diaz-Galvan, Cedres, et al., 2021), while the joint SCD + SOI group might be subclinically associated with other pathologies more strongly linked to olfaction, such as AD (Duff et al., 2002) or Parkinson’s disease (Doty, 2012). We base this speculation on the finding that SCD in the general population is associated with underlying cerebrovascular pathology (Cedres et al., 2019; Diaz-Galvan, Cedres, et al., 2021) and an increased likelihood of progression to vascular dementia (Slot, Sikkes, Berkhof, Brodaty, van der Flier, et al., 2019). Individuals with vascular dementia rarely present olfactory deficits (Duff et al., 2002), since the symptomatology is highly dependent on where the vascular lesions are located (Alves et al., 2014). Future research should address this issue of underlying biological mechanisms, including characterization of joint SCD + SOI individuals and using well-characterized patients in clinical settings.

Interestingly, SOI individuals had a unique emerging pattern of cognitive scores at follow-up. In this group, the odor identification score at follow-up was strongly associated with cognitive performance in memory and visuospatial abilities, even after accounting for the effect of age and education. Furthermore, whereas only the association between memory and visuospatial abilities was significant at baseline; most of the associations at follow-up among the cognitive variables in the SOI group strengthened, becoming significant. Although the correlations between cognition and olfaction were also present in healthy controls, these were weaker, and mostly driven by the higher statistical power for this large subgroup. This finding underscores the relevance of SOI as it may also be associated with future cognitive status. The fact that olfaction and cognition present positive associations reveals that those reporting SOI are at a risk of developing a generalized decline that affects both cognition and olfaction. This pattern was not observed in SCD individuals, where odor identification scores were unrelated to cognitive outcomes at follow-up. The SCD group was unique in that they did not display emerging associations between cognitive abilities. We speculate that these results might point to worse connectivity and brain resilience among SCD. Connected to our previous reasoning, the possibility of underlying cerebrovascular pathology in SCD may be hindering connectivity among brain areas and cognitive processes. In fact, high volumes of white matter lesions have previously been linked to worse white matter integrity (i.e., axonal integrity) in SCD (Diaz-Galvan, Cedres, et al., 2021). Although SCD and SOI might be related to different underlying biological substrates, both conditions have been linked to a future risk of cognitive decline (Ekström et al., 2017; Jonker et al., 2000; Slot, Sikkes, Berkhof, Brodaty, Younsi, et al., 2019). Thus, future studies including disease biomarkers and magnetic resonance imaging could help to determine the shared and unique pathologies underlying these subjective conditions.

In our study, we found that SOI was frequently reported across all age groups, but it was especially frequent in older adults. Age-related olfactory dysfunction is estimated to occur in at least half of the population between ages 65 and 80 (Doty et al., 1984; Murphy et al., 2002). Age-related olfactory dysfunction may have multiple causes, including structural changes in central brain regions for olfactory processing, but also in the olfactory epithelium and bulb (Doty & Kamath, 2014; Doty et al., 1984; Murphy et al., 2002). The emerging association between objective olfaction and cognitive abilities in SOI may point to an underlying pathology targeting medial temporal brain areas; for some individuals, a SOI might be an early step on the path toward a generalized pattern of impaired olfactory and cognitive abilities (Josefsson et al., 2017; Olofsson et al., 2009, 2016; Stanciu et al., 2014). Interestingly, neurodegeneration of medial temporal areas is a hallmark of AD (Ferreira et al., 2020). A recent systematic review and meta-analysis showed an association between AD and decreased volumes of the primary olfactory cortex (Jobin, Boller, et al., 2021). On the other hand, neurodegeneration of primary and secondary olfactory processing areas, including the olfactory bulb (Li et al., 2016) and piriform and orbitofrontal cortex (Lee et al., 2014), have been described in Parkinson’s disease and its associated olfactory decline. Whether the self-perceived changes in olfactory performance in SOI are a harbinger of these underlying neurodegenerative diseases remains unanswered.

The current study has limitations. We lacked enough statistical power to run longitudinal analysis for the combined SCD + SOI individuals. This group showed decreased olfactory performance already at baseline assessment, indicating they are of special interest. Future studies may focus on the characterization of SCD + SOI individuals, as well as on pathological markers such as amyloid, tau, a-synuclein, or underlying cerebrovascular disease that previously have shown associations with self-rated measures (Jylhä et al., 2006). However, it should be noted that in a population-based cohort, the pathological markers are expected to be found at lower levels compared to clinical patients (Kern et al., 2018). Although the presence of allergies was not significantly associated with objective olfaction, the SCD + SOI group showed the highest prevalence compared with the other groups and this may have also influenced the frequency of SOI for this group. Another limitation is that the objective measure used for olfaction is solely based on odor identification. Impaired odor identification is a widely acknowledged feature of AD (Rezek, 1987), but maybe less so for vascular dementia (Motomura & Tomota, 2006). Regarding SCD operationalization, we defined SCD solely based on amnestic complaints. Nevertheless, It has been demonstrated that cognitive complaints referring to different cognitive domains can represent various SCD phenotypes (Diaz-Galvan, Ferreira, et al., 2021). The association between SCD based on nonamnestic complaints and odor identification remains unanswered. Finally, missing data represent a challenge in longitudinal studies. Our longitudinal analysis included 284 individuals. This smaller sample is mostly due to the discontinuation of testing for participants from S2, but there are also missing values at the 10-year follow-up (i.e., 196 participants from S1). This subset was younger and presented higher education level compared with those without follow-up assessment, which may have impacted the results.

This study is, to the best of our knowledge, the first to report and compare the incidence of both SOI and SCD in the general population and investigate long-term cognitive outcomes for these individuals. Results strongly suggest that these reports are complementary and reflect generally independent risk factors. However, when co-occurring, SCD and SOI are associated with objective olfactory impairment and whether this group entails an elevated risk for future dementia remains unanswered. The current study highlights the necessity of including SOI as a valuable screening tool in self-rated assessments for individuals at risk of future olfactory and cognitive decline.
